# 1227. Analysis of Prescriber Interaction with an Electronic Antibiotic Time-out Best Practice Alert

**DOI:** 10.1093/ofid/ofad500.1067

**Published:** 2023-11-27

**Authors:** Tyler Ackley, Anastasia Bilinskaya, Joseph L Kuti, Kristin E Linder, Casey J Dempsey

**Affiliations:** Hartford Hospital, Hartford, Connecticut; Hartford Healthcare, Hartford, Connecticut; Hartford Hospital, Hartford, Connecticut; Hartford HealthCare, Hartford, Connecticut; Hartford Healthcare, Hartford, Connecticut

## Abstract

**Background:**

The impact of antibiotic time-outs (ATOs) on antibiotic use relies heavily on prescriber interaction, which may be reduced due to workflow disruption or alert fatigue. In August 2022, Hartford HealthCare implemented a 72-hour ATO best practice alert. Herein, we evaluated prescriber behavior when interacting with the ATO alert to assess the impact on prescriber stewardship workflow.

**Methods:**

An ATO alert was designed to fire between 0700 and 1630. Upon firing, prescribers were prompted to assess for antibiotic modification – a composite including discontinuation, de-escalation, inclusion of stop-date, and transition to oral therapies. Additional responses were included to defer re-firing for patients deemed not to be a candidate for antibiotic modification. The primary endpoint was defined as the rate of ATO response aligning with antibiotic modification for the 10 most frequently utilized antibiotics and assessed from 10/1/22 – 12/31/22. A predefined subset of patients was evaluated for ATO appropriateness by prescriber specialty and role.

**Results:**

A total of 15,383 ATO alerts were included for analysis, corresponding to 3,498 unique patients. The primary endpoint occurred in 21.4% of cases. The most commonly selected ATO response was a reminder in 10 minutes (48.2%). The median time viewing the ATO alert was 5 seconds (IQR 4 – 8s). In a subset of 400 patients, ATO selections were found to be appropriate in 56% of cases. When stratified by prescriber role, attending physicians were more likely to appropriately utilize the ATO compared to resident and advanced practice practitioners (62.7% vs 48.5% vs 46.2%, p = 0.007) and were more likely to select an ATO response aligning with antibiotic modification (39.5% vs 7.6% vs 12.3%, p < 0.001).

**Conclusion:**

ATO selection corresponding to antibiotic modification was low regardless of prescriber role. Further work optimizing the ATO design will be required to improve integration into stewardship workflow.

**Disclosures:**

**Joseph L. Kuti, PharmD**, bioMeriuex Inc.: Grant/Research Support|Entasis Therapeutics: Grant/Research Support|Merck & Co, Inc: Grant/Research Support|Shionogi Inc: Advisor/Consultant|Shionogi Inc: Grant/Research Support|Shionogi Inc: Honoraria

